# Physical activity to overcome the adversity of widowhood: Benefits beyond physical health

**DOI:** 10.1097/MD.0000000000004413

**Published:** 2016-08-12

**Authors:** Chu-Shiu Li, June Han Lee, Ly-yun Chang, Chwen-Chi Liu, Yan-Lan Chan, Christopher Wen, Mu-Lin Chiu, Min Kuang Tsai, Shan Pou Tsai, Jackson Pui Man Wai, Chwen Keng Tsao, Xifeng Wu, Chi Pang Wen

**Affiliations:** a Department of Risk Management and Insurance, National Kaohsiung First University of Science and Technology, Kaohsiung; b Department of International Business, Asia University, Taichung; c Institute of Population Health Sciences, National Health Research Institutes, Zhunan; d MJ Health Research Foundation, Taipei; e Department of Risk Management and Insurance, Feng Chia University, Taichung, Taiwan; f Department of Radiological Sciences, University of California at Irvine, Irvine, CA; g Department of Public Health, China Medical University, Liaoning; h Institute of Sport Science, National Taiwan Sport University, Taoyuan; i MJ Health Management Institution, Taipei, Taiwan; j Department of Epidemiology, The University of Texas MD Anderson Cancer Center, Houston, TX; k China Medical University Hospital, Taichung and Institute of Population Health Sciences, National Health Research Institutes, Zhunan.

**Keywords:** life expectancy, marital status, physical activity, widow, widowhood

## Abstract

Widowhood has been increasingly encountered because of increasing longevity of women, often characterized by social stigmatization and poor physical and mental health. However, applied research to overcome its adversity has been quite limited. The goal of this study is to explore the role of physical activity in improving the health of widows.

A cohort of 446,582 adults in Taiwan who successively participated in a comprehensive medical screening program starting in 1994, including 232,788 women, was followed up for mortality until 2008. Each individual provided detailed health history, and extensive lab tests results.

The number of widows increased with time trend. Every other woman above age 65 was a widow (44%). Widows were less active, more obese, and smoked and drank more, had sleep problems, were more depressed with taking sedatives or psychoactive drugs, leading to more suicides. In the global development of health policies by World Health Organization (WHO), physical activity is one of the main factors to reverse poor health. The poor health of inactive widow was mitigated when becoming fully active in this study. Exercise not only reduced the observed 18% increase in all-cause mortality, but also gained 4 years and as much as 14% mortality advantage over the married but inactive. More importantly, becoming physically active energized their mental status, improved sleep quality and quantity, reduced depressions and the need for psychoactive drugs, and increased socialization circles.

Widows, a rapidly growing and socially stigmatized group, suffered from social and financial inequality and tended to develop poorer health. Sustained physical activity could be one of the ways for them to overcome and reverse some of the physical and mental adversities of widowhood, and improve their quality and quantity of life.

## Abstract 2

Supplemental Digital Content is available in the text

## 1 Introduction

Widowhood is a dreaded word for all married couples, particularly for the newlyweds, but the reality is that 1 in 2 women above age 65 and 1 in 10 at all ages is expected to become a widow sooner or later, lasting for more than 10 years (see eTable 1 and eTable 2 Supplemental Content, which illustrates the distribution of widows by age in Taiwan) (see eFigure 1 Supplemental Content, which illustrates Taiwan life expectancy gap and first marriage gap between male and female). [^1^ ^2^ ^3^] Asian women have a higher probability of becoming widows, by having a 6.8-year life expectancy advantage, by getting married at an age 3 to 4 years younger than men, and by facing the social reality of difficulty in remarriage after losing the spouse at a later age, when compared to their counterparts in the United States (U.S.) or United Kingdom (UK) (see eTable 2 Supplemental Content, which illustrates the distribution of adults according to marital status in Taiwan, United States, and England and Wales). In Taiwan, the number of widows far outnumbered widowers by up to 6 to 1, with the gender gap increasing with age. The aging of the population accelerated the increase in widows, adding 36% more widows in the last decade, amounting to more than 1 million widows (see eFigure 2 Supplemental Content, which illustrates the rise in the numbers and proportion of widows and widowers in Taiwan). This is a large disadvantaged and stigmatized group in society, and yet their health and welfare have been largely overlooked.

Many widows suffered financial and social loss and changed their lifestyles after bereavement. The downward change in socioeconomic status, the reduction in income and social circles and the limited support available from the society, the dynamic of which was often overlooked, led to poorer health. [^4^ ^5^ ^6^] Some of them were seen to develop drinking habits, take sedatives for stress or become more sedentary, isolated. As a result, widows accelerated decline in physical and mental health. Reports showed increased mortality of widows from all-cause, [^7^ ^8^ ^9^ ^10^ ^11^] cancer,[ ^12^ ^13^] cardiovascular,[ ^14^ ^15^] respiratory diseases, ^[16]^ and others. ^[17]^ Mental illnesses, like depression and suicide, were also higher among widows. [^18^ ^19^ ^20^]

Few women are prepared for the probability of living as a widow for an average of 10 years in Asian countries. The inequality in social status, a key determinant of health, was overlooked by the society. Limited information or research is available to help them cope with the adversity of widowhood. ^[21]^ Only occasionally, referral for counseling has been recommended, mostly limited to serious mental problems.

As physical activity was suggested by WHO as an important factor to reverse the adversity of health,[ ^22^ ^23^] in this study, we examined the effect of physical activity on widows from a large Asian cohort, relative to married women, and compared the long-term outcomes between those physically inactive and active widows. Quantifiable data are available on each individual for their exercise volume, which is a product of exercise intensity and exercise duration, given the previous publications on this subject. ^[24]^ We assessed the possible role of minimal amount of physical activity, such as brisk walking, in modifying or reversing the adverse health outcome expected for widows.

## 2 Materials and methods

### 2.1 Study population and data collection

The total sample contains 446,582 healthy individuals aged 20 years or older, including 16,202 widows and 150,614 married women, who participated in a standard medical screening program run by a private firm between 1996 and 2008. The cohort had 4.5 million person-years of observation, with a median follow-up period of 8.59 years (MJ Health Management Institution, Taipei, Taiwan). A self-administered questionnaire collected information on demographic characteristics, lifestyle, and health risk data. Automated lab tests were given and interpreted at the end of screening appointments in 4 hours by physicians. Informed consent was obtained to authorize the processing and analyzing of the data.

Hypertension was defined as systolic blood pressure ≥140 mm Hg, diastolic ≥90 mm Hg, or the use of hypertension drugs. Diabetes was defined as fasting glucose ≥126 mg/dL, or the use of diabetes drugs. Drinkers were those who indicated drinking 2 drinks 3 times a week or more. Metabolic syndrome was defined by The US National Cholesterol Education Programme Adult Treatment Panel III (NCEP-ATP III) criteria. ^[25]^ By considering the frequency, duration, and intensity reported, the data on physical activity was converted into metabolic equivalent of task (MET)-h/wk, and classified each individual accordingly into “inactive,” “low active,” and “fully active.” “Low active” was equivalent to 15 minutes/d or 90 minutes/wk of moderate intensity activity (3.75–7.49 MET-h/wk), and “fully active” was equivalent to 30 minutes/d or 150 minutes/wk or more (≥7.5 MET-h/wk) of activity. A detailed description of this method has been reported earlier. ^[24]^

### 2.2 Follow-up

All participants in this study signed a consent form and Institutional Review Board (IRB) approval was obtained through the “Research Ethics Committee National Health Research Institutes” (approval number: EC0981201-E) in Taiwan. Individual identification was removed and remained anonymous during the entire study process.

Using unique national identification numbers, deaths and cancer cases in the cohort were ascertained by matching with the National Death File and National Cancer Registry, respectively. A total of 16,849 deaths and 11,802 of cancer cases for the cohort and 1392 deaths for widows were identified.

### 2.3 Statistical analysis

The Cox proportional hazard regression model was used to assess the hazard ratio (HR) and 95% confidence interval (CI) of mortality between marital status and outcomes, compared to those in the reference group. A value of *P* < 0.05 was used for statistical significance. The HRs were adjusted for 10 variables, including 6 continuous and 4 categorical variables. The continuous variables were age, body mass index (BMI), systolic blood pressure, blood glucose, total cholesterol, and urine protein; the categorical variables were education (low education and nonlow education), smoking (nonsmoker, ex-smoker, and current smoker), drinking (never or occasional drinker, and regular drinker) and physical activity (inactive, low active, and fully active). Life expectancy was calculated based on Chiang.[ ^26^ ^27^] Analyses were performed with SAS, version 9.4.

## 3 Results

Widows were less active (47% inactive), more obese, smoked and drank more, and used more sedatives and psychiatric drugs. Widows had higher rates of hypertension, diabetes, and chronic kidney diseases (CDKs), when compared with married women (Table 1).

**Table 1:**
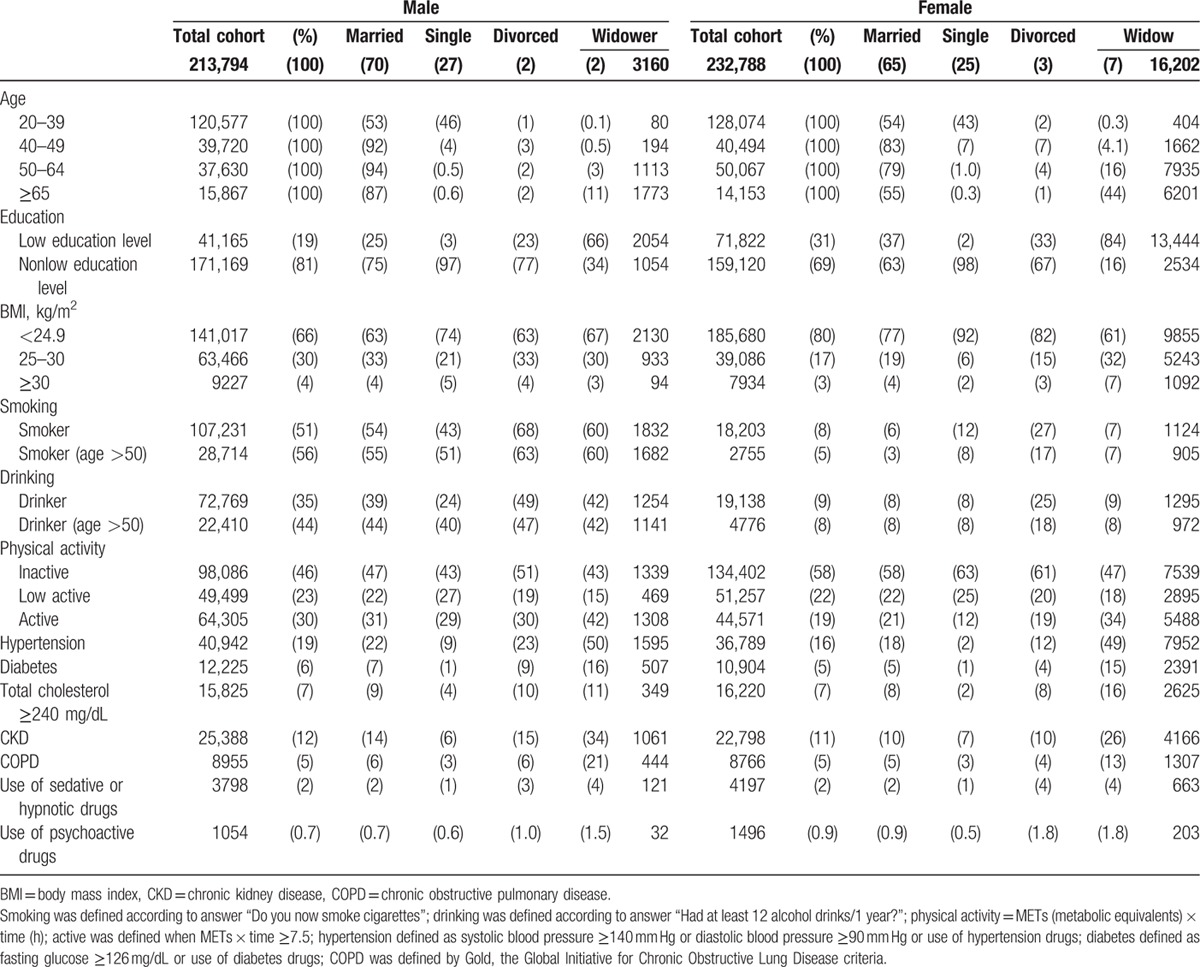
The distribution of the demographic characteristics of the MJ cohort by marital status.

Widows had increased all-cause mortality with adjusted HR at 1.11 (HR: 1.11, 95% CI: 1.03–1.20) or 11% higher than married women. Most causes of death examined for widows in Table 2 show variable increases, but significant increases were only found in respiratory system diseases (HR: 1.64, 95% CI: 1.14–2.36) and chronic obstructive pulmonary disease (COPD) (HR: 1.99, 95% CI: 1.07–3.67). Suicide was also increased, with HR at 1.46, but failed to reach statistical significance.

**Table 2:**
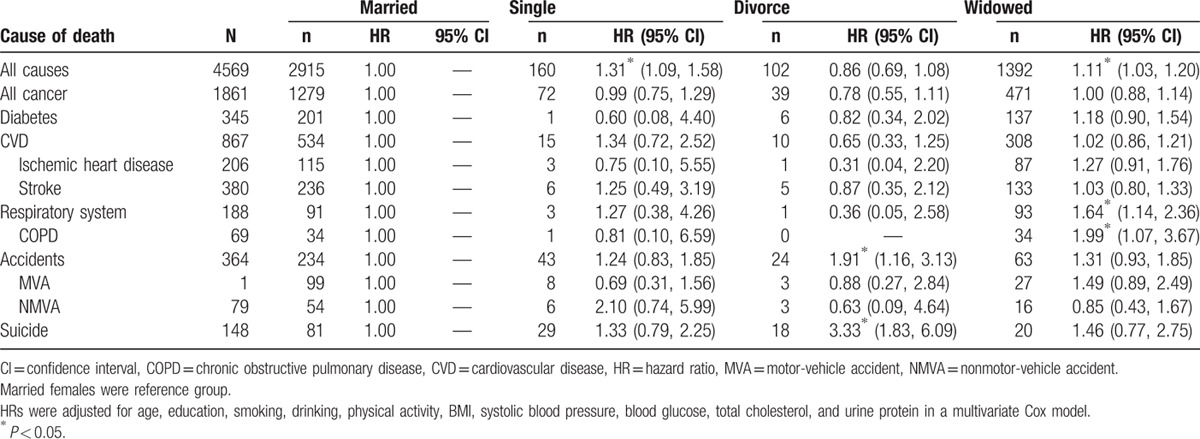
The comparison of mortality risks in females by marital status by different causes of deaths, with married women as the reference.

**Figure 1:**
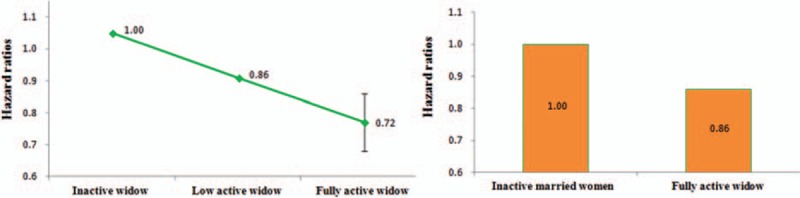
Mortality risk by physical activity status among widow with different reference groups, HRs were adjusted for age, education, smoking, drinking, physical activity, BMI, systolic blood pressure, blood glucose, total cholesterol, and urine protein in a multivariate Cox model.

Table 3 and Fig. 1 show the all-cause mortality by physical activity status. When compared with inactive married women, inactive widows had significant 18% increases (HR: 1.18, 95% CI: 1.06–1.31) in mortality. When widows were physically active, mortality risk reduced and improved to 1.03 (low active) and 0.86 (fully active). The reduction was most significant for the fully active group, but the initially significant increase in mortality for the inactive disappeared for the low active, and became similar to those inactive married women. Further analyses for different age groups are shown in Supplemental Content (see eTable 3 Supplemental Content, which illustrates all-cause mortality risks by marital status and by physical activity status in different age groups for females; see eFigure 3 Supplemental Content, which illustrates forest plot showing decreased mortality risk for active widows among different subgroups). Active widows had similar mortality reduction across age groups and across smoking status.

**Table 3:**
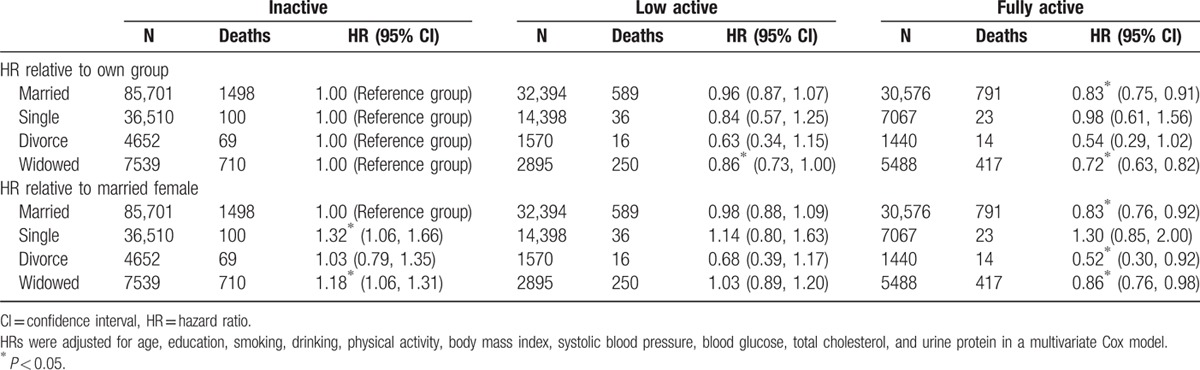
All-cause mortality risks (HR) in females by marital status and by physical activity status with different reference groups.

The quality and quantity of sleep, along with associated mortality risks for problem sleeping, by marital status, are shown in Table 4. There were more <4 and >8 hours of sleep, and more poor quality and needs drug, among inactive widow, when compared against the married. These characteristics of sleep of widow were associated with significantly increased mortality, as shown for the total women cohort. When widow became active, quality and quantity of sleep, such as too short or too long duration, improved.

**Table 4:**
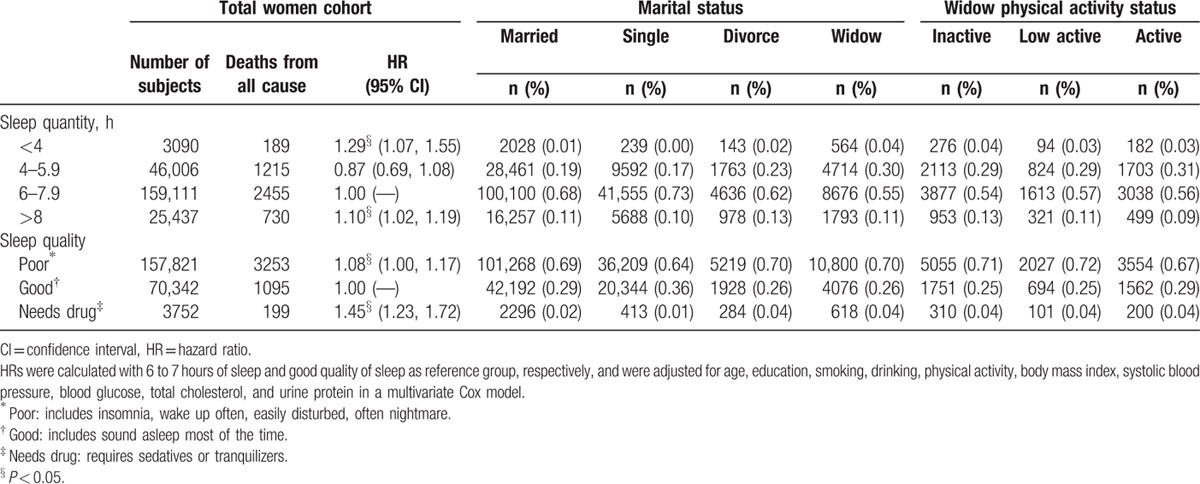
The distribution and hazard ratios for duration and quality of sleep by marital status, with physical activity by sleep duration and quality for the widow.

The benefits of exercise are expressed in life expectancy in Table 5. The life expectancy is shown in Table 5. The top half showed the advantage of physical activity among widows with active widows living 4 years longer than inactive widows. The bottom half showed the differences between active widows and inactive married women with the former living 3.10 years longer than the latter.

**Table 5:**
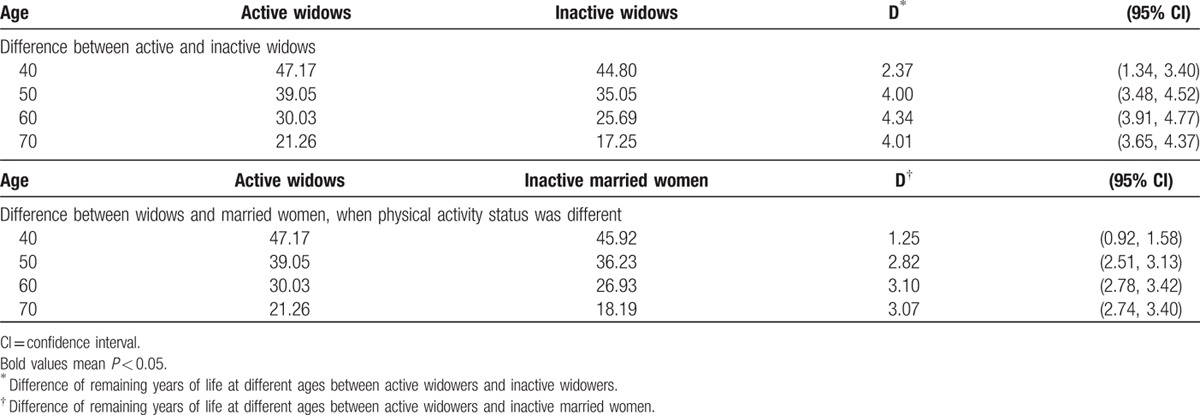
Remaining years in life for widows and for married women by physical activity status.

## 4 Discussion

The number of widows in our society has been increasing rapidly and developing into a potential force with political power, due to increasing longevity, biologic advantages of women, and social disadvantages in their chances of re-marrying. The hardships endured by widows, however, have been overlooked. Both the society and the individual women could have been better prepared for this life-changing event. From the society standpoint, widow can be accorded and recognized as a disadvantage group, requiring financial and social support over and above what little is currently available. From the individual woman's standpoint, early planning before and after widowhood can minimize the hardships, preparing them for the “expected,” normally considered as unexpected. Promoting husband's health, for example, is clearly in the best interest of the spouse. This requires lifelong endeavor to reduce his lifestyle risks and to develop mutually reinforcing exercise habits. To achieve the utopian dream of universal well-being requires a dynamic process of adaptation to a constantly changing environment of a marital life. As change is the only constant that would not change, plasticity of health should be addressed.[ ^28^ ^29^]

Physical activity has been suggested by WHO as a global strategy to improve health of people from all walks of life. ^[23]^ That widows have poorer health and higher mortality than married women has been reported[ ^7^ ^8^ ^10^ ^20^ ^30^ ^31^] but this study demonstrated that, by being physically active, widows could reverse their health adversity and improved not only their physical health but also their mental health. Physically, an active widow extended her life expectancy by 4 years and could outlive a married but inactive woman by up to 3 years, and mentally, exercise improved quality of life by preventing depression and enhancing self-image with expanded socialization. To our knowledge, this is the first report suggesting exercise as a practical modifier to improve the health of a widow's life. Physical activity was beneficial to all walks of life, but regular exercise was far more beneficial to widows than we expected, not only eliminating the 18% mortality disadvantage of the widows, but also reaped an additional 14% mortality advantage when compared to inactive married women, culminating in a combined 32% mortality reduction and extending life expectancy by 3 to 4 years. It is comforting to know that these additional years were spent in active and quality life.

### 4.1 Risk factors and comorbidities

Part of the reasons why widows have increased mortality was due to having more risk factors and comorbidities. In this cohort, as in other studies, widows had more smoking,[ ^32^ ^33^] drinking,[ ^32^ ^34^] obesity,[ ^33^ ^35^ ^36^] sleep problems,[ ^34^ ^37^] hypertension, ^[38]^ and reduced their physical activity ^[39]^ than married women. Either singly or in combination, these risks or comorbidities contributed to poorer health of the widows. However, they could be modified and reduced by increased physical activity, leading to an overall improvement in health.

It is of some interest to assess the timing of these risks, as they could have existed before or acquired after they became widowed. Obviously, both situations could have occurred. In our comparison with married women, we found the health risks of widows quite different from the married, and, in this regard, they must have acquired these habits afterwards (Table 1).[ ^30^ ^40^ ^41^ ^42^] This is consistent with the literature that bereaved women were more likely to pick up drinking[ ^32^ ^34^] and reduce their physical activity, ^[39]^ due in part to an isolated environment and reduced social network.[ ^43^ ^44^] They required more psychoactive drugs to cope with depression. On the other hand, some risks of these widows may have been sustained concurrently with their spouses when they were alive, as “birds of a feather flock together.” In fact, some of these risks could have been associated with the death of their spouse.

### 4.2 Benefits beyond physical health

We listed a few items to illustrate that physical activity was beneficial beyond physical health. First, widow smoked more, drank more, and used more sedatives or psychoactive drugs. Physical activity is known to be beneficial for smokers and for depressed or psychoneurotic subjects. Second, both quality and quantity of sleep by widow were worse than the married and could be an important element in affecting quality of life. We found physical activity had the potential to improve both quality and quantity of sleep. Third, being physically active will facilitate socialization process and improve one's mental health.

### 4.3 Underrated benefits of physical activity

The benefits of exercise for improving physical health have been documented for singles and for the married, for males and for females, for the young and for the old, and for the healthy and for the diseased. ^[24]^ Now we have demonstrated the benefits for the widows. In fact, physical activity has been touted as a miracle drug. ^[45]^ Analysis of this cohort showed, by just brisk walking 15 to 30 minutes a day, average life span can be extended up to 3 years. ^[24]^ At the same time, because physical activity has the advantage of socialization, we have witnessed that exercise cheered people up and reversed the state of isolation and depression.

While widows may have attracted a short-term sympathy from friends and relatives, society as a whole offered very little to them, in areas such as financial, social, or health care support. On the contrary, widows have largely been stigmatized and isolated. In this cohort, widows had poor quality of sleep, drank more and resorted to increased use of sedatives and psychoactive medications, and committed more suicides. While these conditions cannot be changed overnight, they could be mitigated or even reversed by becoming physically active. As we all know, activity could trigger socialization. Exercisers can overcome depression and transform an introverted personality into a more upbeat and sociable attitude and improve emotional quotient (EQ). [^46^ ^47^ ^48^ ^49^ ^50^] It can improve widow's self-image and restore self-confidence, thereby improving both quality and quantity of life. What's more, incorporating regular exercise such as brisk walking for 15 to 30 minutes a day is easy, inexpensive, free from side effects and do-able, for reversing the depression of the widow.

In this study, physical activity was limited to leisure time physical activity (LTPA), 1 of the 4 domains of physical activity.[ ^51^ ^52^] The other 3 domains were transportation-related physical activity, work-related labor, and household activity. LTPA is the only promotable activity most related to health benefits. ^[24]^ The amount of exercise under LTPA would be most effective when a dedicated effort was committed for the sake of health.

Although widows tend to be older and retired, the incomes of widowed women have lagged behind their married counterparts across age groups, with needs-adjusted incomes averaged well-below similarly aged married couples. ^[4]^ The loss of partner's income is particularly substantial, and could have a devastating effect. In addition, evidence suggests their wealth holdings are typically smaller than their married counterparts and cannot offset their lower income. On top of this, the death event may precipitate a large decline in wealth by liquidating assets to cover health care and/or burial costs. While a varying amount of annuity may be added from the death benefits, the data from Taiwan and elsewhere suggest that the gain is likely to be small and is unlikely to alter their social status. The extra-allowance for government employees or labor workers is, at most, 4 to 8 months of salary, earmarked for burial expenses and bereavement benefits.

In Taiwan, the number of widows increased by 36% in 10 years, amounting to more than 1 million in a population of 5.6 million adult women. Nearly half of all Asian women over age 65+ was a widow, a proportion larger than that of the U.S. or UK. While everyone should engage in regular exercise, the message from this study should serve as added motivation for widows to heed and to engage. Parenthetically, encouraging husbands to exercise together, from day 1 and to extend their life span will be equally important so that husbands can also gain a healthier lifestyle and reduce their chance of becoming a widow maker. The policy implications of considering widows as a disadvantaged group and encouraging them to exercise could be profound.

### 4.4 Limitations

There are some limitations worth discussing. First, we only recorded the marital status at the time of first medical screening visit, and did not follow up for subsequent changes in marital status. Because there were more married women becoming widows than widows becoming married, the mortality risks for widows must have been underestimated by not considering those married losing their husband late on. Widows’ benefits of physical activity were also underestimated. Second, since exercise is a desirable behavior, there is a tendency to over-report the level of exercise by everyone. This would inflate the true number of exercisers and lead to underestimating the benefits of physical activity. Third, abundant literature exists supporting the ability of exercise in modifying and improving one's mental health. Limitation of data in this cohort hampered any quantitative assessment of the role of physical activity in improving the mental well-being of widows. However, evidence exists showing the better physical health of those physically active widows are usually associated with better mental health. Fourth, the cohort was all Asians, and the applicability to the whites and blacks is not known. However, previous reports demonstrating similarly increased mortality risks of widows came mainly from the western countries,[ ^20^ ^21^] and the benefits of physical activity were also observed in western countries. ^[52]^

## 5 Conclusion

Widows, suffering from social and financial inequality, had a tendency to develop poorer health. The increased mortality of widows could be mitigated and reversed by physical activity. The physically active widows in our cohort outlived the married but inactive women. Benefits could go beyond physical health, as mental health and quality of life will also be improved. The policy implications to promote exercise can be far reaching.

## Supplementary Material

Supplemental Digital Content

## References

[1] 2014 Statistical Yearbook of Department of Interior: Population by Marital Status 1976–2014; Department of Statistics, Ministry of the Interior, Taiwan. Available at: http://sowf.moi.gov.tw/stat/year/elist.htm. [Accessed 21 June 2016].

[2] 2014 Statistical Yearbook of Department of Interior: Life Expectancy since 1957's; Department of Statistics, Ministry of the Interior, Taiwan. Available at: http://sowf.moi.gov.tw/stat/year/elist.htm [Accessed 21 June 2016].

[3] 2003 Weekly Bulletin of Interior Statistics; Department of Statistics, Ministry of the Interior, Taiwan. Available at: http://www.moi.gov.tw/files/news_file/week10221_1.pdf [Accessed 21 June 2016].

[4] Zick CD Holden K An assessment of the wealth holdings of recent widows. *J Gerontol B Psychol Sci Soc Sci* 2000; 55:S90–S97. 10794193 10.1093/geronb/55.2.s90

[5] Zick CD Smith KR Patterns of economic change surrounding the death of a spouse. *J Gerontol* 1991; 46:S310–S320. 1940097 10.1093/geronj/46.6.s310

[6] Holden KC Smock PJ The economic costs of marital dissolution: why do women bear a disproportionate cost? *Annu Rev Sociol* 1991; 17:51–78. 12285404 10.1146/annurev.so.17.080191.000411

[7] Ytterstad E Brenn T Mortality after the death of a spouse in Norway. *Epidemiology (Cambridge, Mass)* 2015; 26:289–294. 10.1097/EDE.0000000000000266 25695353

[8] Seifter A Singh S McArdle PF Analysis of the bereavement effect after the death of a spouse in the Amish: a population-based retrospective cohort study. *BMJ Open* 2014; 4:e003670. 10.1136/bmjopen-2013-003670 PMC3902313 24435888

[9] Donrovich R Drefahl S Koupil I Early life conditions, partnership histories, and mortality risk for Swedish men and women born 1915–1929. *Soc Sci Med (1982)* 2014; 108:60–67. 10.1016/j.socscimed.2014.02.036 24608121

[10] Berntsen KN Kravdal O The relationship between mortality and time since divorce, widowhood or remarriage in Norway. *Soc Sci Med* 2012; 75:2267–2274. 22995666 10.1016/j.socscimed.2012.08.028

[11] Manzoli L Villari P M Pirone G Marital status and mortality in the elderly: a systematic review and meta-analysis. *Soc Sci Med* 2007; 64:77–94. 17011690 10.1016/j.socscimed.2006.08.031

[12] Va P Yang WS Nechuta S Marital status and mortality among middle age and elderly men and women in urban Shanghai. *PLoS ONE* 2011; 6:e26600. 22073174 10.1371/journal.pone.0026600 PMC3206811

[13] Kravdal H Syse A Changes over time in the effect of marital status on cancer survival. *BMC Public Health* 2011; 11:804. 21999466 10.1186/1471-2458-11-804 PMC3206482

[14] Quinones PA Kirchberger I Heier M Marital status shows a strong protective effect on long-term mortality among first acute myocardial infarction-survivors with diagnosed hyperlipidemia—findings from the MONICA/KORA myocardial infarction registry. *BMC Public Health* 2014; 14:98. 24479754 10.1186/1471-2458-14-98 PMC3937149

[15] Molloy GJ Stamatakis E Randall G Marital status, gender and cardiovascular mortality: behavioural, psychological distress and metabolic explanations. *Soc Sci Med* 2009; 69:223–228. 19501442 10.1016/j.socscimed.2009.05.010 PMC2852675

[16] Berntsen KN Trends in total and cause-specific mortality by marital status among elderly Norwegian men and women. *BMC Public Health* 2011; 11:537. 21733170 10.1186/1471-2458-11-537 PMC3146869

[17] Hadi Khafaji HA Al Habib K Asaad N Marital status and outcome of patients presenting with acute coronary syndrome: an observational report. *Clin Cardiol* 2012; 35:741–748. 22740441 10.1002/clc.22034 PMC6652540

[18] Yan XY Huang SM Huang CQ Marital status and risk for late life depression: a meta-analysis of the published literature. *J Int Med Res* 2011; 39:1142–1154. 21986116 10.1177/147323001103900402

[19] Zhang B Li J Gender and marital status differences in depressive symptoms among elderly adults: the roles of family support and friend support. *Aging Mental Health* 2011; 15:844–854. 21562986 10.1080/13607863.2011.569481

[20] Stroebe M Schut H Stroebe W Health outcomes of bereavement. *Lancet* 2007; 370:1960–1973. 18068517 10.1016/S0140-6736(07)61816-9

[21] Moon JR Kondo N Glymour MM Widowhood and mortality: a meta-analysis. *PLoS ONE* 2011; 6:e23465. 21858130 10.1371/journal.pone.0023465 PMC3157386

[22] Margetts B WHO global strategy on diet, physical activity and health. *Public Health Nutr* 2004; 7:361–363. 15153266 10.1079/PHN2004622

[23] Blair SN Physical inactivity: the biggest public health problem of the 21st century. *Br J Sports Med* 2009; 43:1–2. 19136507

[24] Wen CP Wai JP Tsai MK Minimum amount of physical activity for reduced mortality and extended life expectancy: a prospective cohort study. *Lancet* 2011; 378:1244–1253. 21846575 10.1016/S0140-6736(11)60749-6

[25] National Cholesterol Education Program (NCEP) Expert Panel on Detection, Evaluation, and Treatment of High Blood Cholesterol in Adults (Adult Treatment Panel III). Third Report of the National Cholesterol Education Program (NCEP) Expert Panel on Detection, Evaluation, and Treatment of High Blood Cholesterol in Adults (Adult Treatment Panel III) final report. *Circulation* 2002; 106:3143–3421. 12485966

[26] Wen CP Tsai SP Chung WS A 10-year experience with universal health insurance in Taiwan: measuring changes in health and health disparity. *Ann Intern Med* 2008; 148:258–267. 18283203 10.7326/0003-4819-148-4-200802190-00004

[27] Chiang CL The Life Table and Its Applications. Melbourne, Florida, United States: R.E. Krieger Publishing Company; 1984.

[28] Leischik R Dworrak B Strauss M Plasticity of health. *Ger J Med* 2016; 1:1–17.

[29] Dubos RJ Mirage of Health: Utopias, Progress, and Biological Change. Garden City, NY: Anchor Books; 1959.

[30] Lee S Cho E Grodstein F Effects of marital transitions on changes in dietary and other health behaviours in US women. *Int J Epidemiol* 2005; 34:69–78. 15231759 10.1093/ije/dyh258

[31] Elwert F Christakis NA The effect of widowhood on mortality by the causes of death of both spouses. *Am J Public Health* 2008; 98:2092–2098. 18511733 10.2105/AJPH.2007.114348 PMC2636447

[32] Yim HJ Park HA Kang JH Marital status and health behavior in middle-aged Korean adults. *Korean J Fam Med* 2012; 33:390–397. 23267425 10.4082/kjfm.2012.33.6.390 PMC3526722

[33] Zhang Z Hayward MD Gender, the marital life course, and cardiovascular disease in late midlife. *J Marriage Fam* 2006; 68:639–657.

[34] Doi Y Minowa M Okawa M Prevalence of sleep disturbance and hypnotic medication use in relation to sociodemographic factors in the general Japanese adult population. *J Epidemiol* 2000; 10:79–86. 10778031 10.2188/jea.10.79

[35] Wilson SE Marriage, gender and obesity in later life. *Econ Hum Biol* 2012; 10:431–453. 22795874 10.1016/j.ehb.2012.04.012

[36] Sobal J Rauschenbach B Gender, marital status, and body weight in older U.S. adults. *Gender Issues* 2003; 21:75–94.

[37] Stahl ST Schulz R Changes in routine health behaviors following late-life bereavement: a systematic review. *J Behav Med* 2014; 37:736–755. 23881308 10.1007/s10865-013-9524-7 PMC4197803

[38] Wilcox S Evenson KR Aragaki A The effects of widowhood on physical and mental health, health behaviors, and health outcomes: The Women's Health Initiative. *Health Psychol* 2003; 22:513–522. 14570535 10.1037/0278-6133.22.5.513

[39] Janke MC Nimrod G Kleiber DA Reduction in leisure activity and well-being during the transition to widowhood. *J Women Aging* 2008; 20:83–98. 18581702 10.1300/J074v20n01_07

[40] Roskar S Podlesek A Kuzmanic M Suicide risk and its relationship to change in marital status. *Crisis* 2011; 32:24–30. 21371967 10.1027/0227-5910/a000054

[41] Strohschein L McDonough P Monette G Marital transitions and mental health: are there gender differences in the short-term effects of marital status change? *Soc Sci Med* 2005; 61:2293–2303. 16099576 10.1016/j.socscimed.2005.07.020

[42] Manor O Eisenbach Z Mortality after spousal loss: are there socio-demographic differences? *Soc Sci Med* 2003; 56:405–413. 12473324 10.1016/s0277-9536(02)00046-1

[43] Grimby A Johansson AK Sundh V Walking habits in elderly widows. *Am J Hosp Palliat Care* 2008; 25:81–87. 18160541 10.1177/1049909107307388

[44] Brock AM From wife to widow: a changing lifestyle. *J Gerontol Nurs* 1984; 10:8–11.14–15. 6561227 10.3928/0098-9134-19840401-04

[45] Arem H Moore SC Patel A Leisure time physical activity and mortality: a detailed pooled analysis of the dose-response relationship. *JAMA Intern Med* 2015; 175:959–967. 25844730 10.1001/jamainternmed.2015.0533 PMC4451435

[46] Richards J Jiang X Kelly P Don’t worry, be happy: cross-sectional associations between physical activity and happiness in 15 European countries. *BMC Public Health* 2015; 15:53. 25636787 10.1186/s12889-015-1391-4 PMC4320474

[47] Griffiths A Kouvonen A Pentti J Association of physical activity with future mental health in older, mid-life and younger women. *Eur J Public Health* 2014; 24:813–818. 24532567 10.1093/eurpub/ckt199 PMC4168042

[48] Meyer OL Castro-Schilo L Aguilar-Gaxiola S Determinants of mental health and self-rated health: a model of socioeconomic status, neighborhood safety, and physical activity. *Am J Public Health* 2014; 104:1734–1741. 25033151 10.2105/AJPH.2014.302003 PMC4151927

[49] Mason OJ Holt R Mental health and physical activity interventions: a review of the qualitative literature. *J Mental Health* 2012; 21:274–284. 10.3109/09638237.2011.648344 22533784

[50] Teychenne M Ball K Salmon J Physical activity and likelihood of depression in adults: a review. *Prevent Med* 2008; 46:397–411. 10.1016/j.ypmed.2008.01.009 18289655

[51] Autenrieth CS Baumert J Baumeister SE Association between domains of physical activity and all-cause, cardiovascular and cancer mortality. *Eur J Epidemiol* 2011; 26:91–99. 21153912 10.1007/s10654-010-9517-6

[52] Samitz G Egger M Zwahlen M Domains of physical activity and all-cause mortality: systematic review and dose-response meta-analysis of cohort studies. *Int J Epidemiol* 2011; 40:1382–1400. 22039197 10.1093/ije/dyr112

