# Physical activity to overcome the adversity of widowhood: Benefits beyond physical health: Erratum

**DOI:** 10.1097/01.md.0000505545.78094.f9

**Published:** 2016-10-21

In the article “Physical activity to overcome the adversity of widowhood: Benefits beyond physical health”,^[1]^ which appeared in Volume 95, Issue 32 of *Medicine*, Ly-yun Chan's name was incorrectly included in the author list. The article has since been corrected online.
