# Supplementary material for: Physical activity to overcome the adversity of widowhood: Benefits beyond physical health
Source: Medicine (Baltimore). 2016 Aug 12;95(32):e4413. doi: 10.1097/MD.0000000000004413 (PMC4985311; doi:10.1097/MD.0000000000004413)
Supplement: Supplemental Digital Content [file medi-95-e4413-s001.doc]

**eFigure 1. Taiwan life expectancy gap and first marriage gap between male and female in 2012**


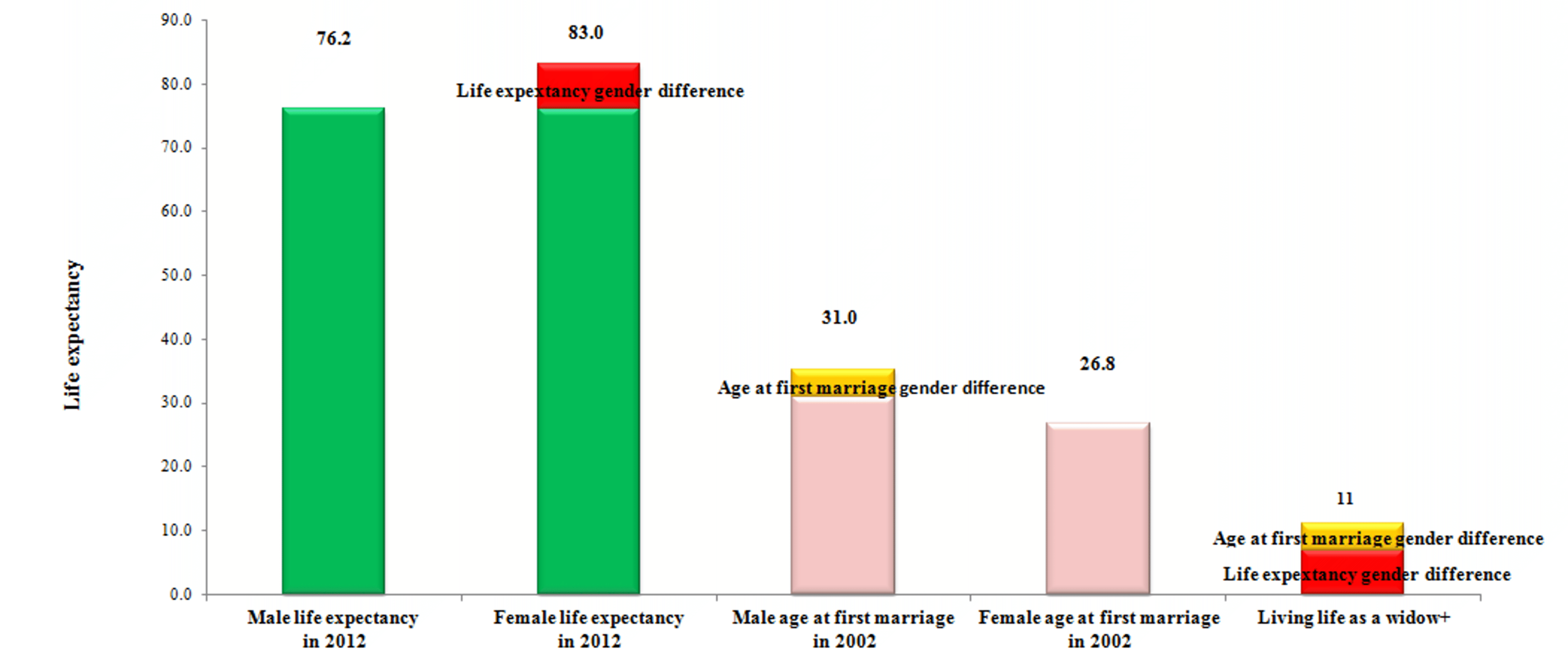


^+^Living life as a widow is the sum of life expectancy gap and age at first marriage gap

^a^ Data source: 2014 Statistical Yearbook of the Department of Interior: Life Expectancy since 1957's; Department of Statistics, Ministry of the Interior, Taiwan. Available at: http://sowf.moi.gov.tw/stat/year/elist.htm and 2003 Weekly Bulletin of Interior Statistics; Department of Statistics, Ministry of the Interior, Taiwan.

Available at: http://www.moi.gov.tw/files/news_file/week10221_1.pdf.

**eFigure 2. The numbers and proportion of widow and widower among all men and women in Taiwan**


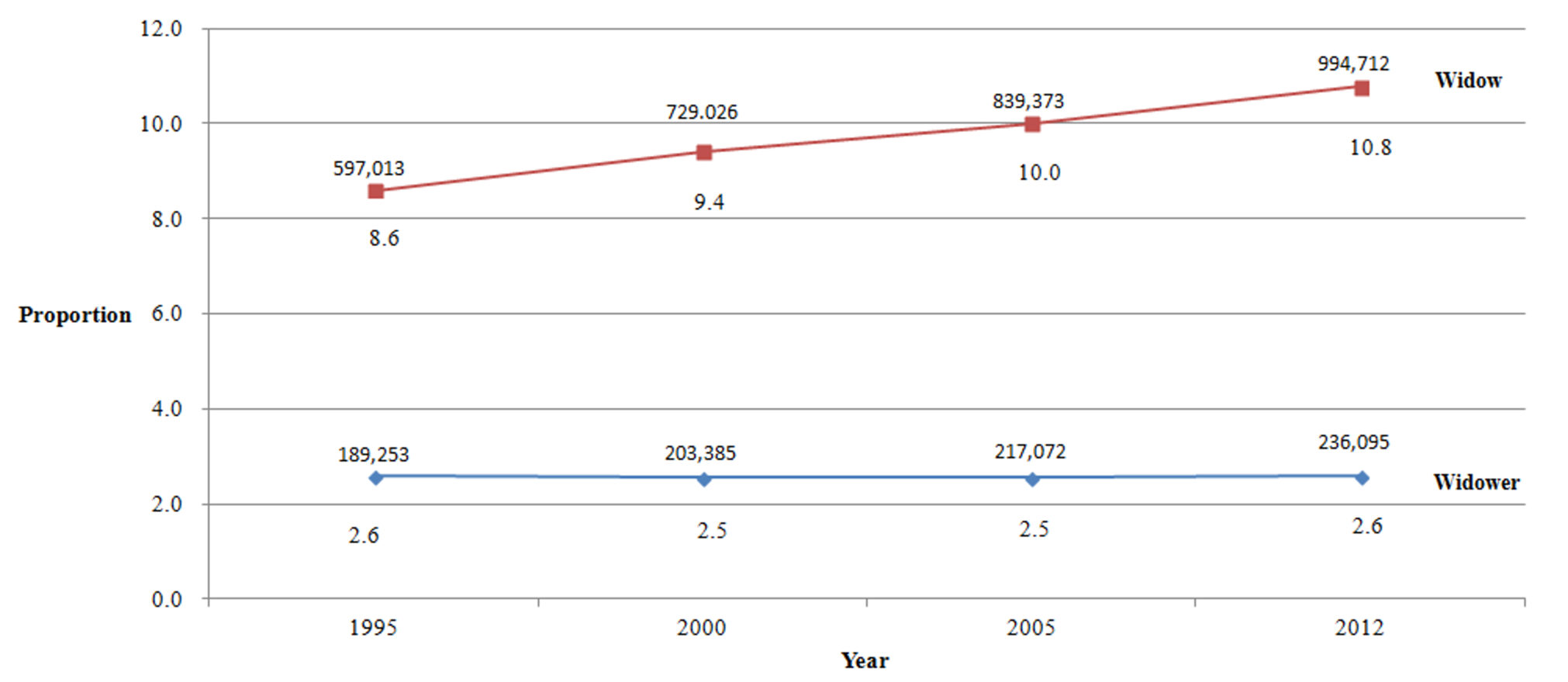


^a^Data source: 2014 Statistical Yearbook of Interior: Population by Marital Status 1976-2014; Department of statistics, Ministry of the interior, Taiwan. Available at: http://sowf.moi.gov.tw/stat/year/elist.htm.

**eFigure 3. Forest plot showing decreased mortality risk for active widows among different subgroups**

**
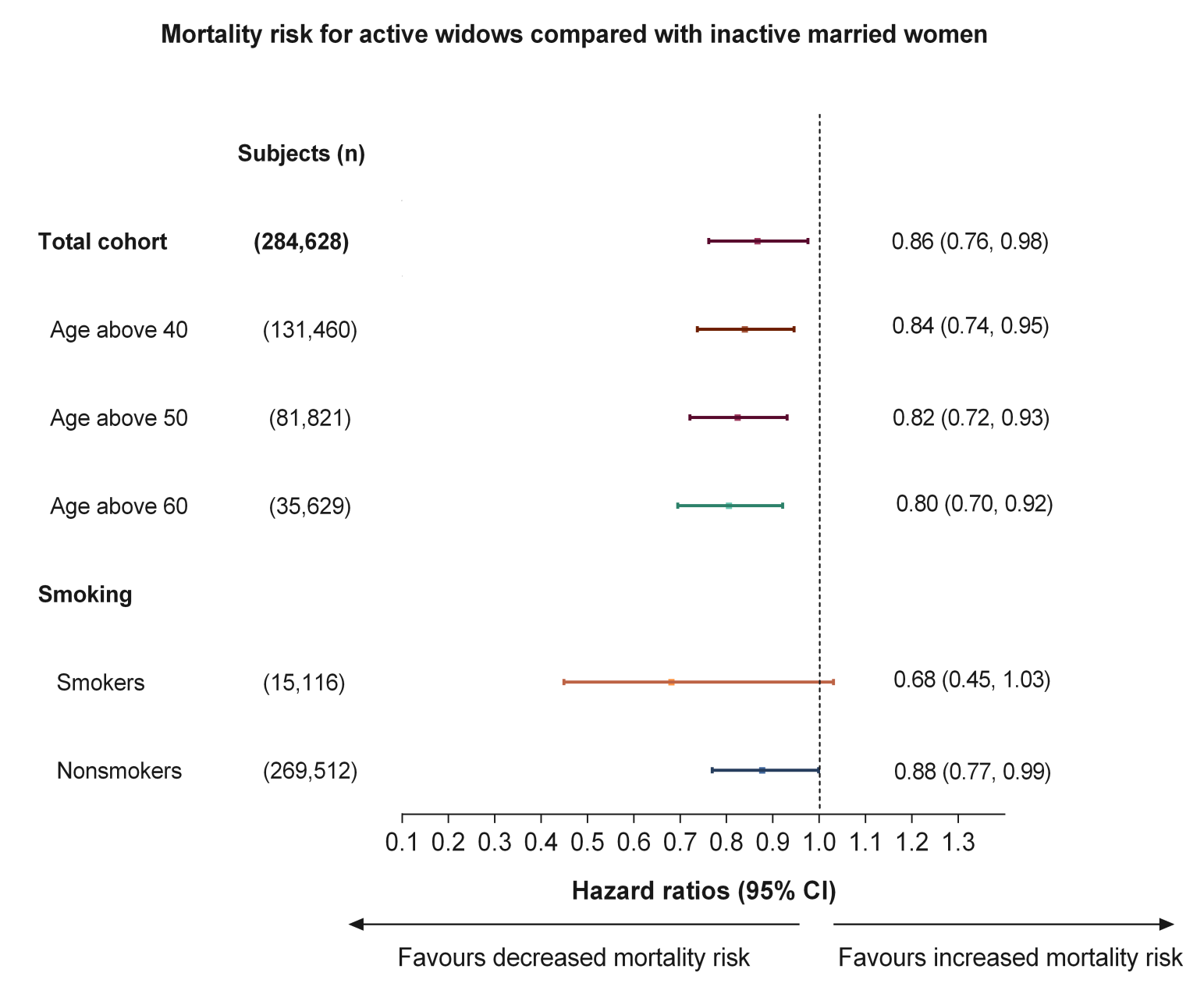
**

^a^Reference group: females who are married

^b^HRs were adjusted for age, education, smoking, drinking, physical activity, BMI, systolic blood pressure, blood glucose, total cholesterol and urine protein in a multivariate Cox model

**eTable 1. Comparison of the distribution of four marital statuses by 10-year and 20-year age groups between males and females for 2012 in the whole Taiwan population**

|  |  | **Male** | | | | | | | | | |  | **Female** | | | | | | | | | |  | **Gender difference** | |
| --- | --- | --- | --- | --- | --- | --- | --- | --- | --- | --- | --- | --- | --- | --- | --- | --- | --- | --- | --- | --- | --- | --- | --- | --- | --- |
| **End of Year & Age** |  | **Total** |  | **Married** |  | **Single** |  | **Divorced** |  | **Widower** | |  | **Total** |  | **Married** |  | **Single** |  | **Divorced** |  | **Widow** | |  | NF-NM | B/A^‡^  ratio |
|  |  | N1 |  | % |  | % |  | % |  | NM | % (A*) |  | N2 |  | % |  | % |  | % |  | NF | % (B^†^) |  |
| 20-29 Years |  | 1,697,980 |  | 7.4 |  | 91.7 |  | 0.8 |  | 83 | 0.005 |  | 1,606,034 |  | 15.2 |  | 82.8 |  | 2.0 |  | 1,142 | 0.1 |  | 1,059 | 15 |
| 30-39 Years |  | 1,944,282 |  | 48.4 |  | 44.8 |  | 6.7 |  | 1,714 | 0.1 |  | 1,969,094 |  | 59.1 |  | 31.4 |  | 8.8 |  | 13,419 | 0.7 |  | 11,705 | 8 |
| 40-49 Years |  | 1,848,198 |  | 70.1 |  | 16.5 |  | 12.9 |  | 8,671 | 0.5 |  | 1,863,961 |  | 70.0 |  | 13.6 |  | 13.5 |  | 52,155 | 2.8 |  | 43,484 | 6 |
| 50-59 Years |  | 1,715,471 |  | 78.0 |  | 7.9 |  | 12.5 |  | 26,820 | 1.6 |  | 1,759,128 |  | 71.6 |  | 6.9 |  | 12.8 |  | 153,778 | 8.7 |  | 126,958 | 6 |
| 60-69 Years |  | 981,289 |  | 83.2 |  | 3.9 |  | 8.7 |  | 41,420 | 4.2 |  | 1,050,511 |  | 66.0 |  | 4.3 |  | 7.3 |  | 235,276 | 22.4 |  | 193,856 | 5 |
| 70+ Years |  | 867,533 |  | 74.5 |  | 3.6 |  | 3.8 |  | 157,387 | 18.1 |  | 985,139 |  | 40.6 |  | 2.3 |  | 2.4 |  | 538,942 | 54.7 |  | 381,555 | 3 |
| 20-39 Years |  | 3,642,262 |  | 29.3 |  | 66.7 |  | 3.9 |  | 1,797 | 0.05 |  | 3,575,128 |  | 39.4 |  | 54.5 |  | 5.7 |  | 14,561 | 0.4 |  | 12,764 | 8 |
| 40-59 Years |  | 3,563,669 |  | 73.9 |  | 12.4 |  | 12.7 |  | 35,491 | 1.0 |  | 3,623,089 |  | 70.8 |  | 10.4 |  | 13.2 |  | 205,933 | 5.7 |  | 170,442 | 6 |
| 60+ Years |  | 1,848,822 |  | 79.1 |  | 3.8 |  | 6.4 |  | 198,807 | 10.8 |  | 2,035,650 |  | 53.7 |  | 3.3 |  | 4.9 |  | 774,218 | 38.0 |  | 575,411 | 4 |
| 20+Total |  | 9,054,753 |  | 57.0 |  | 32.6 |  | 7.9 |  | 236,095 | 2.6 |  | 9,233,867 |  | 54.9 |  | 25.9 |  | 8.5 |  | 994,712 | 10.8 |  | 758,617 | 4 |

*A=NM/N1. ^†^ B=NF/N2. ^‡^ B/A=(NF/N1)/(NM/N2).

Abbreviations: N1, number of male; NM, number of widower; N2, number of female; NM, number of widow.

Data source: 2014 Statistical Yearbook of the Department of Interior: Population by Marital Status 1976-2014; Department of Statistics, Ministry of the Interior, Taiwan. Available at: http://sowf.moi.gov.tw/stat/year/elist.htm.

**eTable 2. Distribution of adults according to marital status, Taiwan, United States and England and Wales**

|  | | **Taiwan*** | | | | | | | | | | |  | **United States^†^** | | | | | | | | | | |  | **England and Wales^‡^** | | | | | | | | | |
| --- | --- | --- | --- | --- | --- | --- | --- | --- | --- | --- | --- | --- | --- | --- | --- | --- | --- | --- | --- | --- | --- | --- | --- | --- | --- | --- | --- | --- | --- | --- | --- | --- | --- | --- | --- |
|  | | Male (N=9,054,753) | | | | |  | Female (N=9,233,867) | | | | |  | Male (N=108,139,000) | | | | |  | Female (N=114,837,0000) | | | | |  | Male (N=20,702,518) | | | | |  | Female (N=21,942,871) | | | |
|  | | n | | (%) | | |  | n | | (%) | | |  | n | | (%) | | |  | n | | (%) | | |  | n | | (%) | | |  | n | | (%) | |
| **Age 40-64** | |  | |  | | |  |  | |  | | |  |  | |  | | |  |  | |  | | |  |  | |  | | |  |  | |  | |
| Married | | 3152087 | | (75.3) | | |  | 3012178 | | (70.3) | | |  | 35055000 | | (70.7) | | |  | 35049000 | | (67.5) | | |  | 5755970 | | (63.4) | | |  | 5731430 | | (61.7) | |
| Single | | 467086 | | (11.2) | | |  | 407727 | | (9.5) | | |  | 6683000 | | (13.5) | | |  | 5670000 | | (10.9) | | |  | 1976109 | | (21.8) | | |  | 1609485 | | (17.3) | |
| Divorce | | 512260 | | (12.2) | | |  | 533394 | | (12.5) | | |  | 7156000 | | (14.4) | | |  | 8589000 | | (16.6) | | |  | 1217601 | | (13.4) | | |  | 1583748 | | (17.1) | |
| Widowed | | 56689 | | (1.4) | | |  | 329657 | | (7.7) | | |  | 698000 | | (1.4) | | |  | 2589000 | | (5.0) | | |  | 124834 | | (1.4) | | |  | 363209 | | (3.9) | |
| **Age 65+** | | | | | | | | | | | | | | | | | | | | | | | | | | | | | | | | | | |  |
| Married | 945576 | | (77.2) | |  | 645949 | | | (47.0) | |  | 12530000 | | | (73.4) | |  | 10095000 | | | (45.7) | |  | 2846832 | | | (69.5) | |  | 2309534 | | | (45.0) | |  |
| Single | 43045 | | (3.5) | |  | 35263 | | | (2.6) | |  | 760000 | | | (4.5) | |  | 948000 | | | (4.3) | |  | 320503 | | | (7.8) | |  | 297772 | | | (5.8) | |  |
| Divorce | 58139 | | (4.7) | |  | 44077 | | | (3.2) | |  | 1637000 | | | (9.6) | |  | 2508000 | | | (11.4) | |  | 337817 | | | (8.2) | |  | 460609 | | | (9.0) | |  |
| Widowed | 177609 | | (14.5) | |  | 650494 | | | (47.3) | |  | 2149000 | | | (12.6) | |  | 8526000 | | | (38.6) | |  | 591009 | | | (14.4) | |  | 2058997 | | | (40.2) | |  |

*Data source: 2014 Statistical Yearbook of the Department of Interior: Population by Marital Status 1976-2014; Department of Statistics, Ministry of the Interior, Taiwan. Available at: http://sowf.moi.gov.tw/stat/year/elist.htm.

^†^From United States Census Bureau: America’s Families and Living Arrangements: 2011;Reported "Married Spouse Present", "Married Spouse Absent" and "separated" were categorized into "Married".

^‡^From UK office for National Statistics: How have Living Arrangements and Marital Status in England and Wales Changed Since 2001? ; "Married/civil partnered" were categorized into "Married"; "Single" and "Separated" were categorized into "Single".

**eTable 3. All-cause mortality risks by marital status and by physical activity status in different age groups for females**

|  | **Total** | | | | | | | **Inactive** | | | | | | | | **Low Active** | | | | | | | | **Fully Active** | | | | | | | |
| --- | --- | --- | --- | --- | --- | --- | --- | --- | --- | --- | --- | --- | --- | --- | --- | --- | --- | --- | --- | --- | --- | --- | --- | --- | --- | --- | --- | --- | --- | --- | --- |
|  | N | Deaths | HR |  | 95%CI | |  | | N | Deaths | HR |  | 95%CI | |  | | N | Deaths | HR |  | 95%CI | |  | | N | Deaths | HR |  | 95%CI | |  |
| **Age ≧40** |  |  |  |  |  |  |  | |  |  |  |  |  |  |  | |  |  |  |  |  |  |  | |  |  |  |  |  |  |  |
| Married | 79540 | 2633 | 1.00 |  | - | |  | | 39258 | 1343 | 1.00 |  | - | |  | | 16589 | 524 | 0.96 |  | (0.86 | ,1.07) |  | | 23693 | 766 | 0.82 | # | (0.74 | ,0.91) |  |
| Single | 3146 | 37 | 1.26 |  | (0.89 | ,1.79) |  | | 1718 | 25 | 1.39 |  | (0.90 | ,2.15) |  | | 761 | 5 | 0.81 |  | (0.34 | ,1.96) |  | | 667 | 7 | 1.14 |  | (0.54 | ,2.40) |  |
| Divorce | 4710 | 77 | 0.74 | # | (0.57 | ,0.96) |  | | 2657 | 53 | 0.87 |  | (0.63 | ,1.19) |  | | 943 | 12 | 0.54 |  | (0.28 | ,1.05) |  | | 1110 | 12 | 0.46 | # | (0.25 | ,0.86) |  |
| Widowed | 15523 | 1371 | 1.08 | * | (1.00 | ,1.17) |  | | 7267 | 708 | 1.15 | * | (1.03 | ,1.28) |  | | 2819 | 247 | 0.99 |  | (0.86 | ,1.16) |  | | 5437 | 416 | 0.84 | # | (0.74 | ,0.95) |  |
| **Age ≧50** |  |  |  |  |  |  |  | |  |  |  |  |  |  |  | |  |  |  |  |  |  |  | |  |  |  |  |  |  |  |
| Married | 46584 | 2295 | 1.00 |  | - | |  | | 21152 | 1142 | 1.00 |  | - | |  | | 8967 | 457 | 0.97 |  | (0.86 | ,1.10) |  | | 16465 | 696 | 0.82 | # | (0.73 | ,0.91) |  |
| Single | 511 | 15 | 1.00 |  | (0.58 | ,1.74) |  | | 237 | 9 | 0.93 |  | (0.44 | ,1.97) |  | | 116 | 1 | 0.38 |  | (0.05 | ,2.70) |  | | 158 | 5 | 1.45 |  | (0.60 | ,3.50) |  |
| Divorce | 2038 | 48 | 0.65 | # | (0.47 | ,0.90) |  | | 1051 | 31 | 0.70 |  | (0.46 | ,1.06) |  | | 384 | 7 | 0.60 |  | (0.29 | ,1.26) |  | | 603 | 10 | 0.47 | # | (0.23 | ,0.94) |  |
| Widowed | 13887 | 1351 | 1.08 |  | (0.99 | ,1.17) |  | | 6258 | 700 | 1.15 | * | (1.03 | ,1.29) |  | | 2522 | 243 | 0.99 |  | (0.85 | ,1.15) |  | | 5107 | 408 | 0.82 | # | (0.72 | ,0.93) |  |
| **Age ≧60** |  |  |  |  |  |  |  | |  |  |  |  |  |  |  | |  |  |  |  |  |  |  | |  |  |  |  |  |  |  |
| Married | 16985 | 1490 | 1.00 |  | - | |  | | 7109 | 722 | 1.00 |  | - | |  | | 3075 | 300 | 0.99 |  | (0.85 | ,1.15) |  | | 6801 | 468 | 0.78 | # | (0.69 | ,0.89) |  |
| Single | 95 | 11 | 1.09 |  | (0.56 | ,2.10) |  | | 44 | 7 | 1.02 |  | (0.42 | ,2.46) |  | | 24 | 1 | 0.58 |  | (0.08 | ,4.09) |  | | 27 | 3 | 1.50 |  | (0.48 | ,4.67) |  |
| Divorce | 464 | 19 | 0.44 | # | (0.25 | ,0.76) |  | | 217 | 15 | 0.65 |  | (0.35 | ,1.21) |  | | 81 | 2 | 0.32 |  | (0.08 | ,1.28) |  | | 166 | 2 | 0.10 | # | (0.01 | ,0.70) |  |
| Widowed | 9090 | 1192 | 1.09 |  | (0.99 | ,1.19) |  | | 3902 | 612 | 1.16 | * | (1.02 | ,1.31) |  | | 1586 | 216 | 1.01 |  | (0.85 | ,1.19) |  | | 3602 | 364 | 0.80 | # | (0.70 | ,0.92) |  |

^a^Reference group: females who are married

^b^HRs were adjusted for age, education, smoking, drinking, physical activity, BMI, systolic blood pressure, blood glucose, total cholesterol and urine protein in a multivariate Cox model

*indicates a significantly (*p*<0.05) higher risk compared to the reference group

#indicates a significantly (*p*<0.05) lower risk compared to the reference group
